# Characterization of a practically designed plastic scintillation plate dosimeter

**DOI:** 10.1002/mp.17904

**Published:** 2025-05-31

**Authors:** Takeshi Ohta, Yuki Nozawa, Shingo Ohira, Kanabu Nawa, Hideomi Yamashita, Keiichi Nakagawa

**Affiliations:** ^1^ Department of Radiology The University of Tokyo Hospital Tokyo Japan; ^2^ Department of Comprehensive Radiation Oncology The University of Tokyo Tokyo Japan; ^3^ Department of Radiological Sciences Faculty of Health Sciences Tokyo Metropolitan University Tokyo Japan; ^4^ Kansai BNCT Medical Center Osaka Medical and Pharmaceutical University Osaka Japan

**Keywords:** characteristics, CMOS camera, plastic scintillator, scintillator plate, x‐ray beams

## Abstract

**Background:**

Advancements in radiotherapy have enabled the use of high‐definition irradiation, leading to more precise and finely adjusted treatments in clinical settings. Nevertheless, the attainment of high resolution, an extensive measurement area, and the repeatability of dose distribution measurements persist as challenges in clinical practice, thereby often requiring multiple dosimetry systems to overcome measurement constraints. Consequently, there is a significant need to develop a dosimeter that offers both a high resolution and a capability for repeated use.

**Purpose:**

A practical scintillation plate dosimeter was designed and its dosimetric characteristics were evaluated using x‐ray beams from a linear accelerator.

**Methods:**

A practical scintillation plate dosimeter comprised a 0.2 cm‐thick scintillation plate sandwiched between a pair of 2.0 cm‐thick Polymethyl methacrylate (PMMA) plates. A Complementary Metal Oxide Semiconductor (CMOS) camera was used to detect the scintillation light emitted from the scintillation plate when the x‐ray beams were delivered to the plate. Measurements were made at 6 MV to test the dose linearity, reproducibility, and dependencies on the camera temperature and angles of incidence. The dose‐rate dependency was also measured using 6 and 10 MV flattening filter‐free (FFF) beams. The x‐ray energy dependency was further tested using 4 MV, 6 MV, 10 MV, 6 MV FFF, and 10 MV FFF beams.

**Results:**

A maximum linearity error of 0.4% was observed for doses ranging from 10 to 1000 MU. The coefficient of variation for the dose reproducibility was ± 0.062%, the temperature dependency was 0.07%/°C, and the angular variations were within ± 1.3% after the removal of Cherenkov light. The dose output decreased by 5.0% at 45 MU/min, compared with that at 1300 MU/min with the 6 MV FFF beams, and by 2.0% at 160 MU/min, compared to 1900 MU/min with the 10 MV FFF beams. The dependency of x‐ray energy ranged from −2.1% to +1.4%.

**Conclusions:**

The practical scintillation plate dosimeter showed favorable dose characteristics that can be applied in patient‐specific quality assurance for volumetric modulated arc therapy.

## 1 INTRODUCTION

Scintillation dosimetry has a long history. Beddar et al.^1^, ^2^ published the first systematic study of plastic scintillation detectors (PSDs) for high‐energy beam dosimetry in 1992. Since then, the PSD has been increasingly studied in x‐ray and electron radiotherapy. In 2007, Beddar^3^ published a review article on PSD, in which many favorable characteristics were restated with measured data, including water equivalence, energy independence, good linearity and reproducibility, and little temperature dependence. In 2016, inspired by more recent requirements for PSD application to multi‐dimensional real‐time scintillation dosimetry for radiotherapy, including proton therapy, Beaulieu and Beddar^4^ published a topical review article on PSD. In this review, several studies using plastic scintillator sheets were reported, and their high spatial resolution was highlighted, compared with that of array‐detector dosimeters used for quality assurance (QA). In 2006, a study on a tissue‐equivalent plastic scintillator‐based dosimetry system demonstrated its effectiveness in verifying two‐dimensional dose distributions. The evaluation of the prototype system was promising, confirming that the average pixel intensity increased linearly with the administered dose.^5^ In 2008, a verification study was conducted on the 2D scintillation dosimeter DosiMap. This device is unique in that it employs two innovative methods, the subtraction method and the colorimetric method, to separate Cherenkov radiation and scintillation. The study compared the accuracy, precision, and spatial resolution of each method.^6^ In 2022, a further imaging study for dose QA was published using 3D printed scintillator arrays.^7^

To the best of our knowledge, scintillation imaging systems for patient QA in Volumetric Modulated Arc Therapy (VMAT) are not commercially available. This is most likely due to technical difficulties in optical elements of a scintillation imaging device that meet specifications for accuracy and reliability comparable to those of other commercial‐dose QA systems. Under these circumstances, we developed a scintillation plate dosimeter for practical applications. The developed scintillation dosimeter is designed to have a high spatial resolution (0.0369 cm/pixel) and a large measurement area (24 × 24 cm^2^). The mating parts of the camera unit and scintillation unit have the same shape and are interchangeable. By swapping the camera unit and the scintillation unit, both axial and coronal dose distributions can be measured.

The dosimeter has been in clinical use at the University of Tokyo Hospital since April 2023. Over the past 1.5 years, 1000 VMAT verifications have been performed. Development of machine QA and beam QA protocols is currently underway.

The purpose of this study was to evaluate the essential characteristics of the developed scintillation plate dosimeter and to compare it with the reported values of commercially available array detectors, including MapCHECK,^8^, ^9^, ^10^, ^11^ ArcCHECK,^12^, ^13^ Delta4,^14^, ^15^ and MatriXX.^16^, ^17^

## 2 MATERIALS AND METHODS

### 2.1 Device description

**FIGURE 1:**
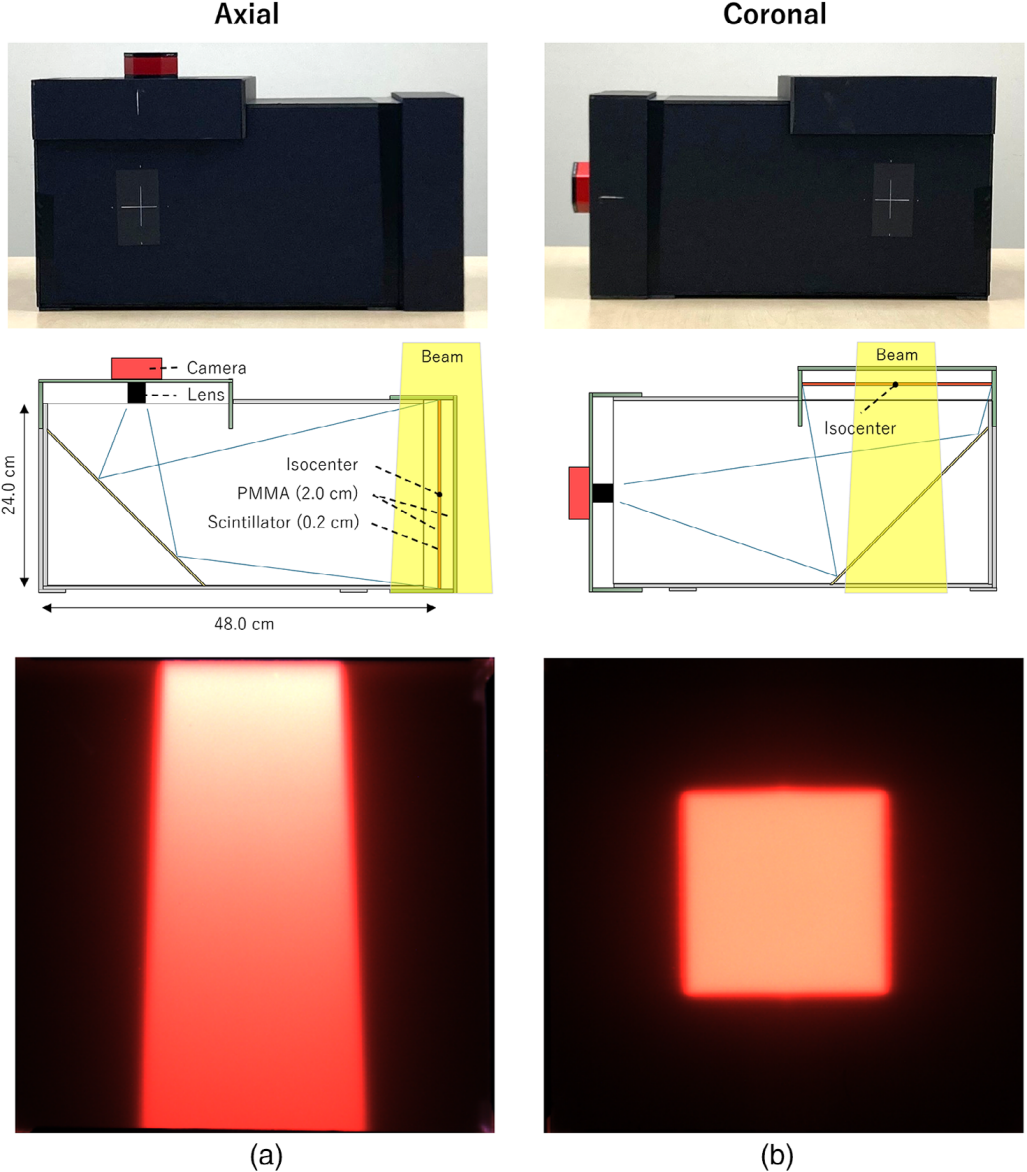
(a) Structure of the plastic scintillation plate dosimeter for the measurement and an example of light distribution obtained by camera on an axial plane. (b) That is for the measurement on a coronal plane. The measurement plane can be changed by swapping the scintillation and camera units.

Figure 1a,b shows the structure of the scintillation plate dosimeter used for measurements in the axial and coronal planes. The plastic scintillator is a novel design and composed of polystyrene doped with 1% Diphenyl oxazole (DPO) and 0.08% red wavelength shifter (610 nm). The physical density and electron density of the scintillator are 1.05 ± 0.01 g/cc and 1.02 ± 0.01 g/cc, respectively. The scintillator plate was positioned between two transparent Polymethyl methacrylate (PMMA) buildup layers, each 2.0 cm thick, resulting in a total assembly thickness of 4.2 cm. All measurements and evaluations were performed by placing the center of the scintillation plate at the isocenter (a source‐to‐detector distance of 1 m) in the axial or coronal plane. The housing was composed of a 0.5 cm‐thick polystyrene board with a density of 0.1 g/cc. A mirror was placed inside the assembly to change the direction of scintillation light propagation and direct it toward the camera.

A Complementary Metal Oxide Semiconductor (CMOS) camera was used to detect the scintillation light emitted from the scintillation plate when the x‐ray beams were delivered to the plate. Both dark noise and readout noise are important for continuous imaging when using a scintillator. After comparing many industrial cameras, a CMOS planetary RGB color camera, namely, Uranus‐C (Player One Astronomy, Suzhou, China) with a Sony image sensor IMX585, was selected. IMX585 has a dark noise of 0.7e‐ and read noise of 1e‐. The image matrix was configured to 2000 × 2000 pixels using an in‐house driver for the camera. In the current configuration, 650 × 650 pixels cover the entire scintillation plate, resulting in a pixel size of 0.0369 × 0.0369 cm^2^ in the scintillation detector plane.

The plastic scintillator was manufactured from a single plate rather than a discrete array, producing a smooth and continuous dose distribution. The hardware and software were developed in an integrated manner, with optical challenges addressed primarily on the hardware side.

Both sides of the acrylic sheets are treated with an anti‐reflection coating to suppress unwanted reflections. Furthermore, the scintillator surface is coated with an anti‐glare treatment to promote the efficient emission of scintillation light. Although the two plates are not optically bonded and an air layer is present between them, these surface treatments minimize optical impedance loss. The mirror is made of resin and has aluminum vapor deposition on the front surface, rather than the back surface. The use of a front‐surface reflective type prevents the generation of double ghost images. These combined strategies achieve a high‐contrast dose distribution without blurring. This approach minimized the need for complex software corrections, thereby reducing processing load.

Imaging, image processing, and analysis are performed by application software. The application software was created using the LabVIEW software (National Instruments, Austin, USA). The images are acquired in color, and only the red channel image is extracted. The red channel image, which has a dynamic range of 12 bits, is accumulated as a 16‐bit integer image. Both vignetting correction caused by the lens and spike noise reduction using 3 × 3 median filtering are performed in real‐time during imaging. In this study, images were acquired at 5 frames per second (FPS), but the system can operate at higher frame rates. Once the shooting was complete, the Cherenkov light component was removed from the accumulated Image and converted linearly into a dose distribution.

### 2.2 Cherenkov separation

About 5%–10% of the signal in the red channel is Cherenkov light, while the rest is scintillation light. The Cherenkov ratio varies with x‐ray incident angles and increases in proportion to the beam path length of the acrylic plate during VMAT delivery. By separating the scintillation light and the Cherenkov light, the relatively large angular dependence of the Cherenkov light contribution is suppressed, allowing a more accurate measurement of the dose distribution. To separate these two lights, the following equations are used for the RGB channels.

(1) $$\;T_{R}=S_{R}+C_{R}$$

(2) $$\;T_{G}=S_{G}+C_{G}$$

(3) $$\;T_{B}=S_{B}+C_{B}$$

where *T*, *S*, and *C* denote the light intensities of the total, scintillation, and Cherenkov components, respectively.

Multiply both sides of Equation (2) by *C_B_* and both sides of Equation (3) by *C_G_*.

(4) $$T_{G}\;C_{B}=S_{G}\;C_{B}+C_{G}C_{B}$$

(5) $$T_{B}\;C_{G}=S_{B}\;C_{G}+C_{B}C_{G}$$

Subtracting Equations (5) from (4) gives the following values for *S_R_*.

(6) $$\;S_{R}=\frac{T_{G}-T_{B}\frac{C_{G}}{C_{B}}}{\frac{S_{G}}{S_{R}}-\frac{S_{B}}{S_{R}}\frac{C_{G}}{C_{B}}}\;$$

Then multiply both sides of Equation (2) by *S_B_* and both sides of Equation (3) by *S_G_*.

(7) $$T_{G}\;S_{B}=S_{G}\;S_{B}+C_{G}S_{B}$$

(8) $$T_{B}\;S_{G}=S_{B}\;S_{G}+C_{B}S_{G}$$

Subtracting Equations (8) from (7) gives the following values for *C_R_*.

(9) $$\;C_{R}=\frac{T_{G}-T_{B}\frac{S_{G}}{S_{B}}}{\frac{C_{G}}{C_{R}}-\frac{C_{B}}{C_{R}}\frac{S_{G}}{S_{B}}}\;$$

where ratios such as *S_B_*/*S_R_* and *C_B_*/*C_G_* represent the pre‐measured luminance ratios of scintillation and Cherenkov light, which are intrinsic values independent of the beam's incident angle or acrylic thickness. The RGB luminance ratio was obtained by irradiating the beam before assembling the scintillator and acrylic plates. Solving the last equations pixel by pixel accounts for variations in the Cherenkov ratio. A similar formula appears in reference of the DosiMap,^6^ differing in form but equivalent in principle, with the same calibration procedure. The luminance (or luminance ratios) of Cherenkov and scintillation light in the RGB channels are pre‐measured.

### 2.3 Evaluations

For evaluating linearity, reproducibility, dose‐rate dependence, temperature dependence, angular dependence, and energy dependence, a central 10 × 10 pixels area (0.369 × 0.369 cm^2)^ was designated as the region of interest (ROI), serving as a representative value for camera output. A 3 × 3 pixels area (0.1 × 0.1 cm) is susceptible to statistical fluctuations, and it is possible that the measurement results cannot be evaluated quantitatively. Therefore, the side length was set to 10 pixels.

For evaluating angular dependence and VMAT QA, the process of removing Cherenkov light generated from PMMA was performed. In other evaluation tests, the irradiation direction is constant, so quantitative evaluation is possible even without correction.

To eliminate the effects of changes in the state of the linear accelerator, all property evaluation measurements were carried out on the same day.

#### 2.3.1 Dose comparison between measurements and calculations

This device is intended for use in IMRT/VMAT QA. The first test of our plastic scintillation plate dosimeter is dose distribution comparisons between measurements and calculations by delivering a 10 × 10 cm^2^ beam having a photon energy of 6 MV, where Monaco 5.1 (Elekta AB, Stockholm, Sweden) was used as a treatment planning system (TPS). The density was replaced by the density of acrylic and scintillator.

#### 2.3.2 Linearity

Next, the dose linearity was evaluated by delivering 6 MV x‐ray beams with a field size of 10 × 10 cm^2^ for doses ranging from 1 to 1000 MU using an Elekta VersaHD linac (Elekta AB, Stockholm, Sweden). The scintillation plate was set on the coronal plane at the isocenter, and the beam was delivered to the plate at a gantry angle of 0°. The central 10 **× **10 pixel (0.369 × 0.369 cm^2^) intensities on the camera image plane were averaged for this test.

#### 2.3.3 Reproducibility

The dose reproducibility was also measured by delivering 100 MU 10 times in reference to simultaneously acquired ionization chamber readings using a Semiflex chamber 31010 (PTW, Freiburg, Germany). The other measurement parameters were identical to those used in the linearity tests.

#### 2.3.4 Dose‐rate dependence

The dose‐rate dependence was then examined. The aimed dose rates were varied between 50 and 2400 MU/min, and a dose of 100 MU was delivered using 6 and 10 MV flattening‐filter‐free (FFF) x‐ray beams. The other measurement parameters were identical to those used in the linearity test.

#### 2.3.5 Temperature dependence

Subsequently, the camera‐temperature dependence was evaluated after turning on the camera, and the camera temperature was monitored using a temperature sensor integrated inside the camera. We wanted to examine temperature dependence because the camera is not always on, and for QA, waiting for it to reach thermal equilibrium prior to measurement is not practical. A dose of 100 MU was repeatedly delivered to the scintillation plate with an x‐ray energy of 6 MV until the temperature reached equilibrium with the built‐in, air‐cooled heat sink. The other measurement parameters were identical to those used in the linearity tests.

#### 2.3.6 Angular dependence

The angular dependence of incident beams was also measured by changing the gantry angle from −180° to 180° with a step size of 30°, each with a delivered dose of 100 MU. The measured value was normalized by dividing it by the calculated dose on the scintillation plate at each incident angle to compensate for different absorptions in the 2 cm‐thick PMMA plate, with a dose grid size of 0.2 cm and an uncertainty of 0.5%. To avoid couch attenuation influence, the scintillation plate dosimeter (Figure 1) was flipped over for the measurements at gantry angles greater than 90° and less than −90°.

#### 2.3.7 Energy dependence

Then, the x‐ray energy dependence was inspected by providing five energies: 4 MV, 6 MV, 10 MV, 6 MV FFF, and 10 MV FFF, each with a delivered dose of 100 MU. The camera output was normalized by dividing it by the calculated dose on the scintillation plate for each energy to compensate for the different absorptions in the 2 cm‐thick PMMA plate. The other measurement parameters were identical to those used in the linearity tests.

#### 2.3.8 VMAT QA

Finally, dose distributions from a clinical lung stereotactic VMAT plan were compared between our dosimeter and the TPS calculation. The plan consisted of a single arc with counterclockwise gantry rotation from 179° to 181°, delivering a total of 3240 MU using a 6 MV FFF beam.

## 3 RESULTS

### 3.1 Dose comparison between measurements and calculations

**FIGURE 2:**
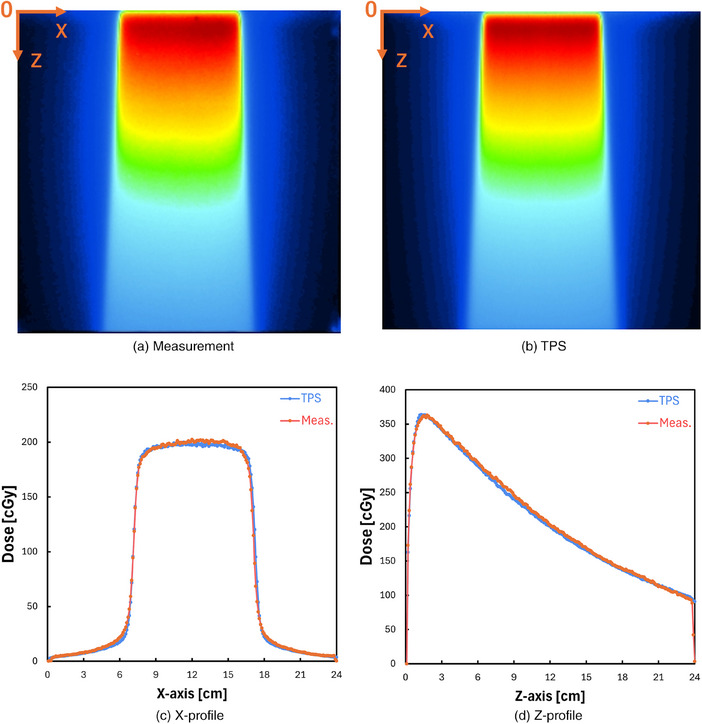
(a) An example dose distribution of an x‐ray beam measured on the axial plane. (b) An example dose distribution of an x‐ray beam planned on the axial plane. (c) *X*‐axis dose profile across the center of the scintillation plate. (d) *Z*‐axis dose profile across the center of the scintillation plate.

Figure 2a,b shows measured and planned dose distributions on an axial plane for a 6 MV beam having a field size of 10 × 10 cm^2^, respectively. Figure 2c,d compares measured and planned dose distributions in the lateral (*X*‐axis) and the depth (*Z*‐axis) directions, respectively. The 2D gamma passing rate by using gamma analysis criteria of 3%/2 mm, 2%/2 mm, and 1%/1 mm of dose distance/distance‐to‐agreement and 10% for the threshold was 99.57%, 98.74%, and 88.96%, respectively.

**FIGURE 3:**
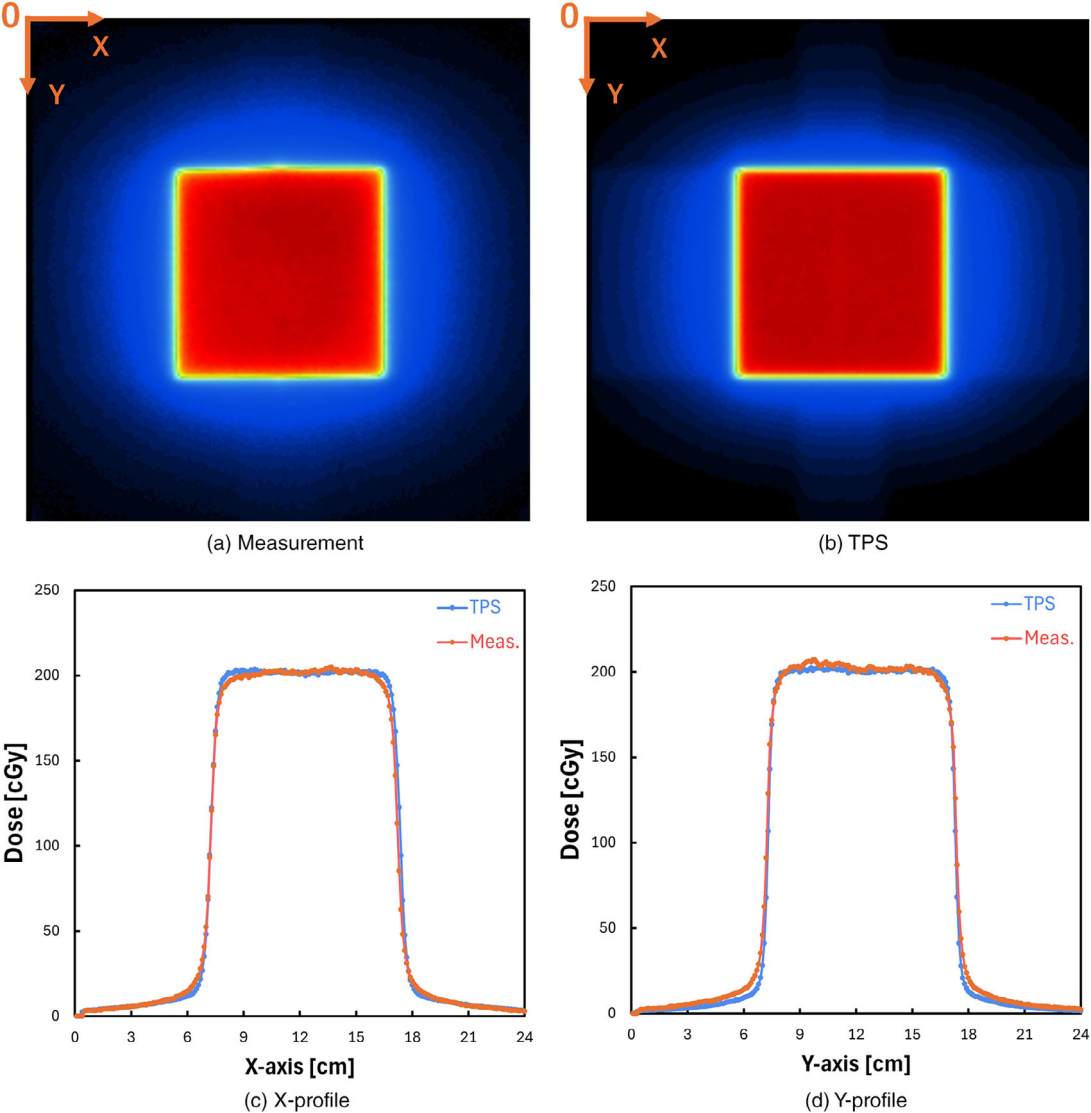
(a) An example dose distribution of an x‐ray beam measured on the coronal plane. (b) An example dose distribution of an x‐ray beam planned on the coronal plane. (c) *X*‐axis dose profile across the center. (d) *Y*‐axis dose profile across the center.

Figure 3a,b shows measured and planned dose distributions on a coronal plane for a 6 MV beam having a field size of 10 × 10 cm^2^, respectively. Figure 3c,d compares measured and planned dose distributions in the *X* and *Y* directions, respectively. Again, the measured and calculated results showed good agreement. The 2D gamma passing rates by using gamma analysis criteria of 3%/2 mm, 2%/2 mm, and 1%/1 mm of dose distance/distance‐to‐agreement and 10% for the threshold were observed to be 98.08%, 92.35%, and 62.62%, respectively.

### 3.2 Linearity

**FIGURE 4:**
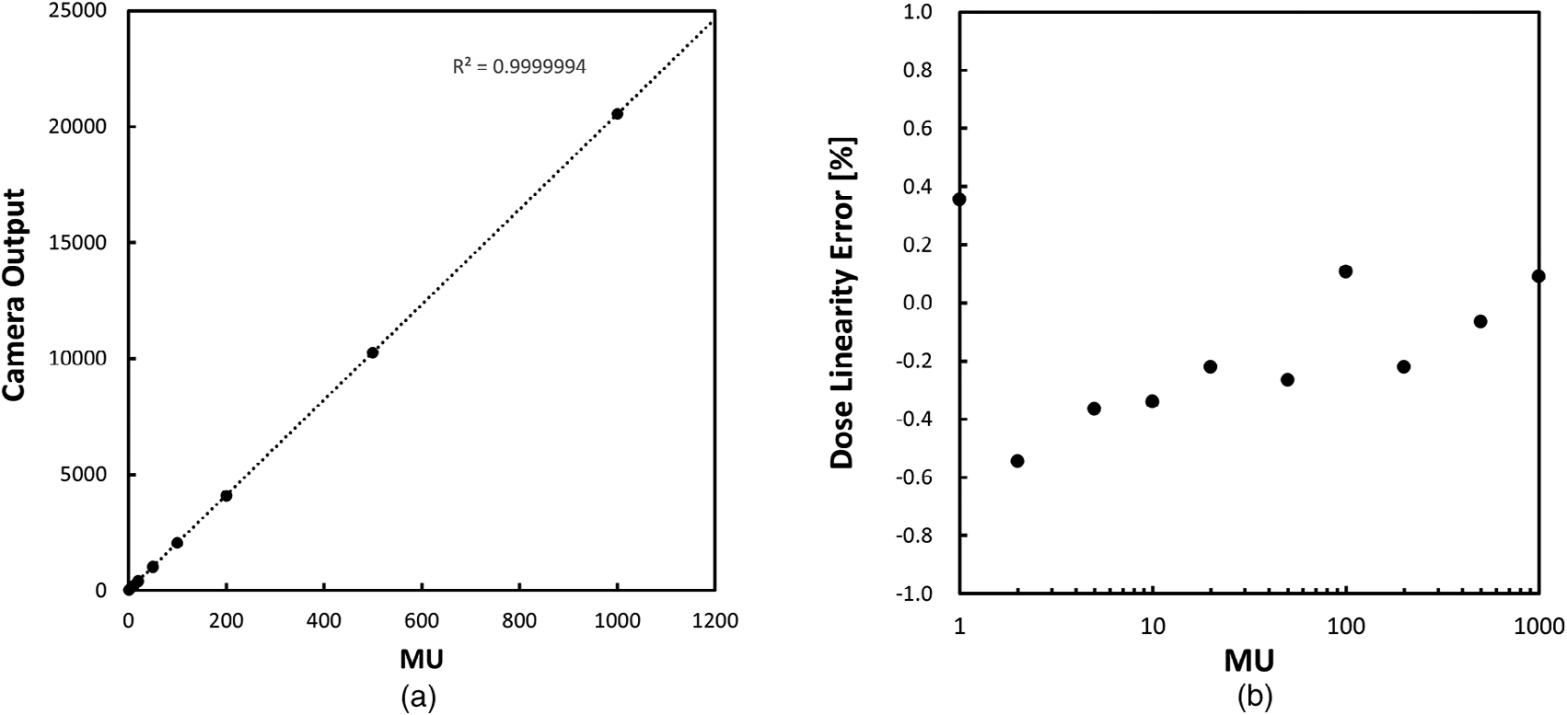
(a) Plot of the camera output signal as a function of the delivered dose for a field size of 10 × 10 cm^2^ with an x‐ray energy of 6 MV. (b) Plot of the resulting dose linearity error as a function of the delivered dose. The scintillation plate was set on the coronal plane at the isocenter, and the pixel intensities on the 10 × 10 pixels at the image center were averaged for this test.

Figure 4a shows the camera output as a function of the delivered MU for a field size of 10 × 10 cm^2^ and an x‐ray energy of 6 MV. Figure 4b shows the dose linearity error as a function of MU. A maximum linearity error of 0.6% was observed for doses ranging from 1 to 1000 MU, and a maximum linearity error of 0.4% was observed for doses ranging from 10 to 1000 MU. The actual radiation dose was equivalent to 0.937 cGy per 1 MU, with the center of the scintillator as the isocenter.

### 3.3 Reproducibility

**FIGURE 5:**
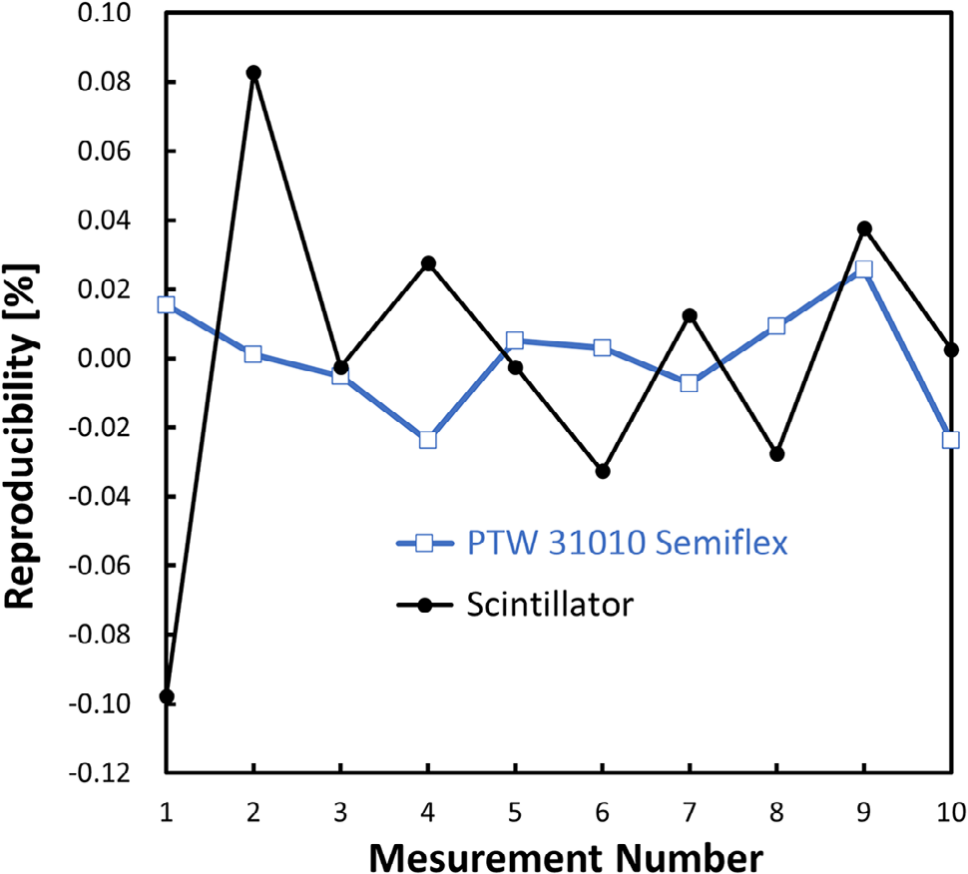
Plot of the reproducibility of camera outputs by delivering 100 MU 10 times for a field size of 10 × 10 cm^2^ with an x‐ray energy of 6 MV. The scintillation plate was set on the coronal plane at the isocenter, and the pixel intensities of the 10 × 10 pixels at the image center were averaged. A Semiflex chamber was also placed for comparison.

Figure 5 shows the reproducibility of the camera outputs after delivering 100 MU 10 times. The coefficients of variation for the camera outputs and chamber readings were 0.062% and 0.016%, respectively.

### 3.4 Dose‐rate dependence

**FIGURE 6:**
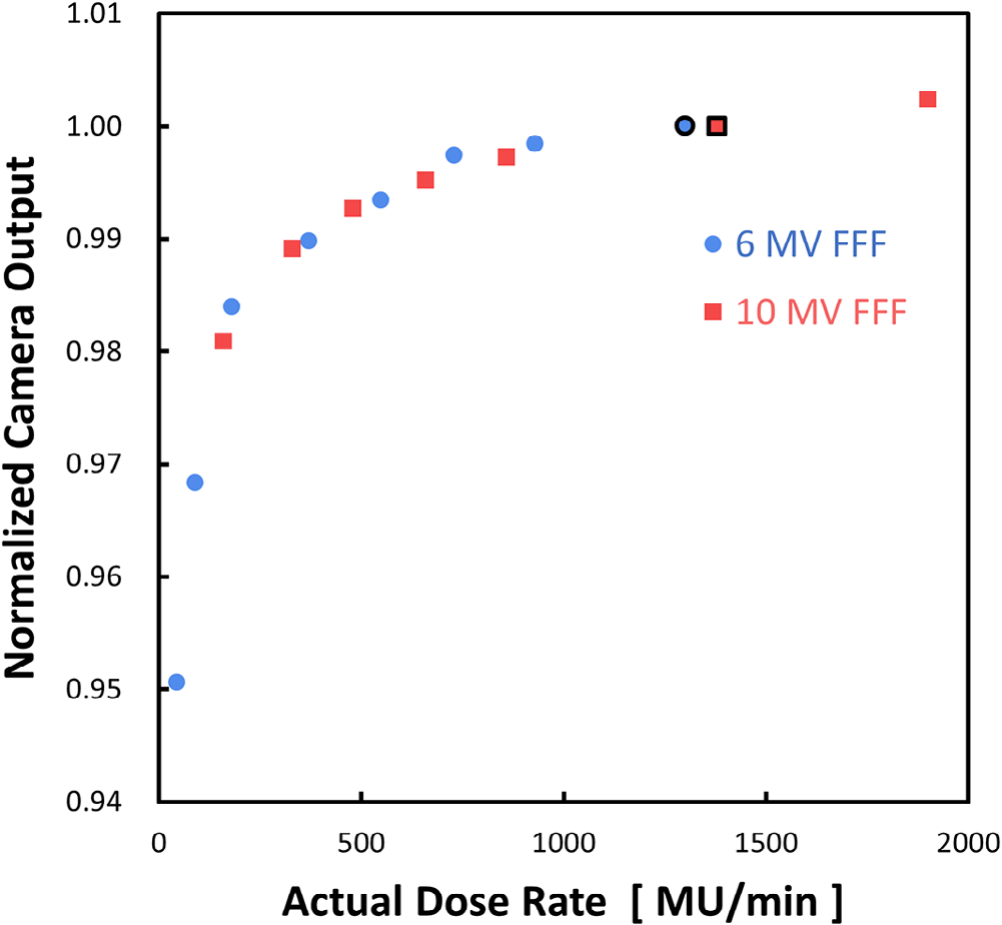
Plot of the normalized camera outputs as a function of the actual dose rates ranging from 45 to 1900 MU/min. The normalization was made against the black‐bordered points. A dose of 100 MU was delivered with 6 and 10 MV flattening‐filter‐free (FFF) x‐ray beams with a field size of 10 × 10 cm^2^. The scintillation plate was set on the coronal plane at the isocenter, and the pixel intensities on the 10 × 10 pixels at the image center were averaged for this test.

Figure 6 shows the camera outputs as a function of the actual dose rates, which differ from the aimed dose rates, owing to the characteristics of the Elekta linacs. The camera outputs decreased by 5.0% at 45 MU/min, compared with those at 1300 MU/min with 6 MV FFF beams, and by 2.0% at 160 MU/min, compared with those at 1900 MU/min with 10 MV FFF beams.

### 3.5 Temperature dependence

**FIGURE 7:**
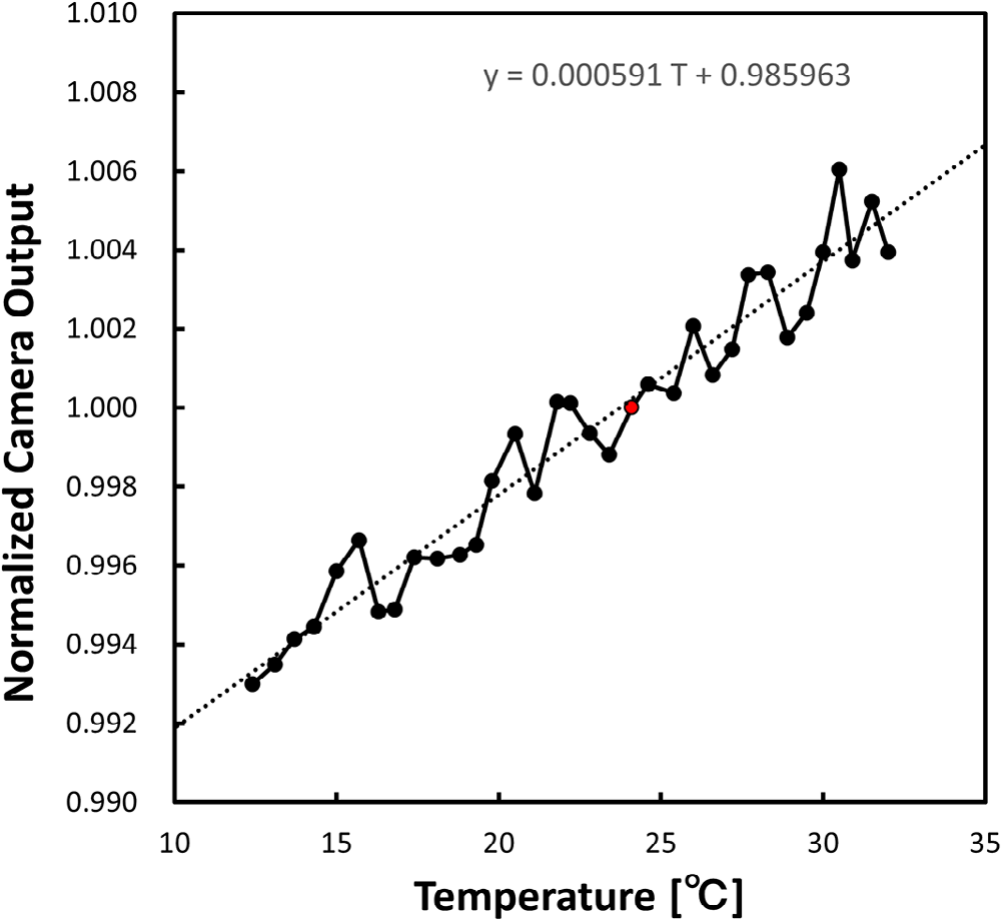
Plot of the normalized camera output as a function of the camera temperature. The normalization was made at 24.1°C, which is one of the measurement points closest to room temperature. This is illustrated by the red circle. The camera temperature was monitored by a temperature sensor integrated inside the camera. During this test, a dose of 100 MU was repeatedly delivered to the scintillation plate with an x‐ray energy of 6 MV. The scintillation plate was set on the coronal plane at the isocenter, and the pixel intensities of the 10 × 10 pixels at the image center were averaged.

Figure 7 shows the camera output as a function of the camera temperature when a dose of 100 MU was repeatedly delivered to the scintillation plate. The temperature of the CMOS sensor changes from room temperature to over 30°C in about 10 min. For this evaluation, the sensor was cooled using an ice pack to obtain a wide temperature range in the camera output. At most, the camera output linearly increased when the temperature changed from 12.4° to 32.0° in 2 h, and the temperature dependence was approximately 0.06%/°C. The regression line for the temperature *T* and camera output was (5.91 × 10^−4^)T + 0.986. The reference value was selected as 24.1°C, which is the measurement point closest to normal room temperature. It takes 1 h for the camera to reach 30°C from the room temperature of 24°C, but in the assumed use of patient‐specific QA, the work is completed before the camera reaches equilibrium temperature.

### 3.6 Angular dependence

**FIGURE 8:**
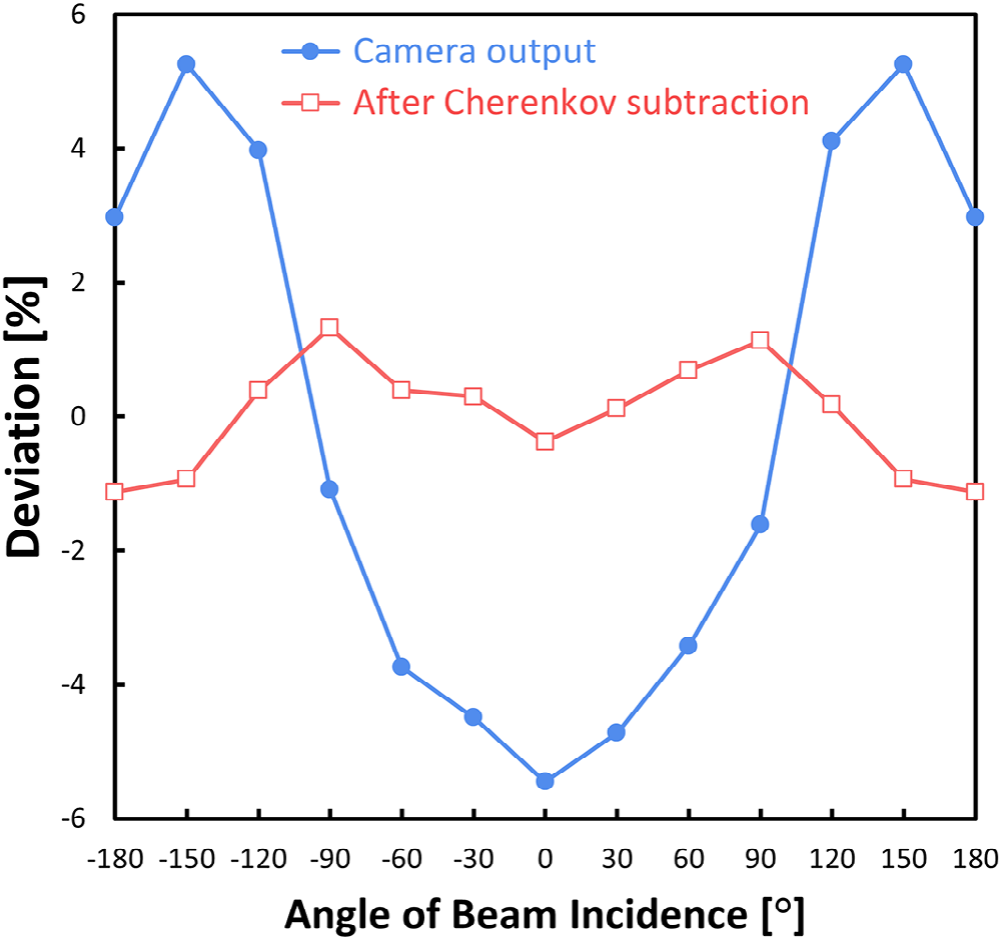
Plot of the camera output deviations as a function of the angle of incident x‐ray beams before and after Cherenkov background subtraction. The measured value was normalized by dividing it by the planned dose on the scintillation plate at each angle to compensate for different absorption values in the 2 cm‐thick PMMA plate. Then, the deviations were plotted so that the mean deviation of each plot was zero. A dose of 100 MU was delivered with a photon energy of 6 MV and a field size of 10 × 10 cm^2^. The scintillation plate was set on the coronal plane at the isocenter, and the pixel intensities on the 10 × 10 pixels at the image center were averaged for this test.

Figure 8 shows the camera output as a function of the angle of the incident x‐ray beam. After the Cherenkov light image was subtracted from the measured image, the angular deviations were reduced to approximately ±1.3%.

### 3.7 Energy dependence

Table 1 lists the calculated doses obtained using the TPS for each photon energy. The scintillation plate was set on the coronal plane at the isocenter, and pixel intensities of the 10 × 10 pixels at the image center were averaged for this test. The camera output was normalized by dividing it by the calculated dose on the scintillator for each photon energy with a field size of 10 × 10 cm^2^ and a delivered dose of 100 MU.

**TABLE 1:** Energy dependence of the camera output, normalized by dividing it by the calculated dose.

Photon energy	4 MV	6 MV	6 MV FFF	10 MV	10 MV FFF
Normalized camera output	0.979	1.0000	0.993	1.014	1.005
TPR_20,10_	0.637	0.6808	0.6756	0.7333	0.7189

**FIGURE 9:**
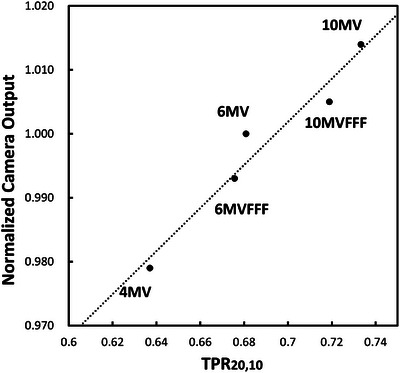
The relationship between TPR_20,10_ and normalized camera outputs. TPR_20,10_ is measured value in the water phantom.

Figure 9 shows the relationship between the energy and the output value of the camera, using the energy as a quality factor (TPR_20,10_). Figure 9 shows the relationship between the TPR_20,10_ and the normalized camera output. TPR_20,10_ values were obtained by measuring the dose at depth of 20 and 10 cm with a water phantom. The trend between the TPR_20,10_ and the camera output demonstrates a linear response, indicating that an energy correction factor for the camera output can be given by the TPR_20,10_.

### 3.8 VMAT QA

**FIGURE 10:**
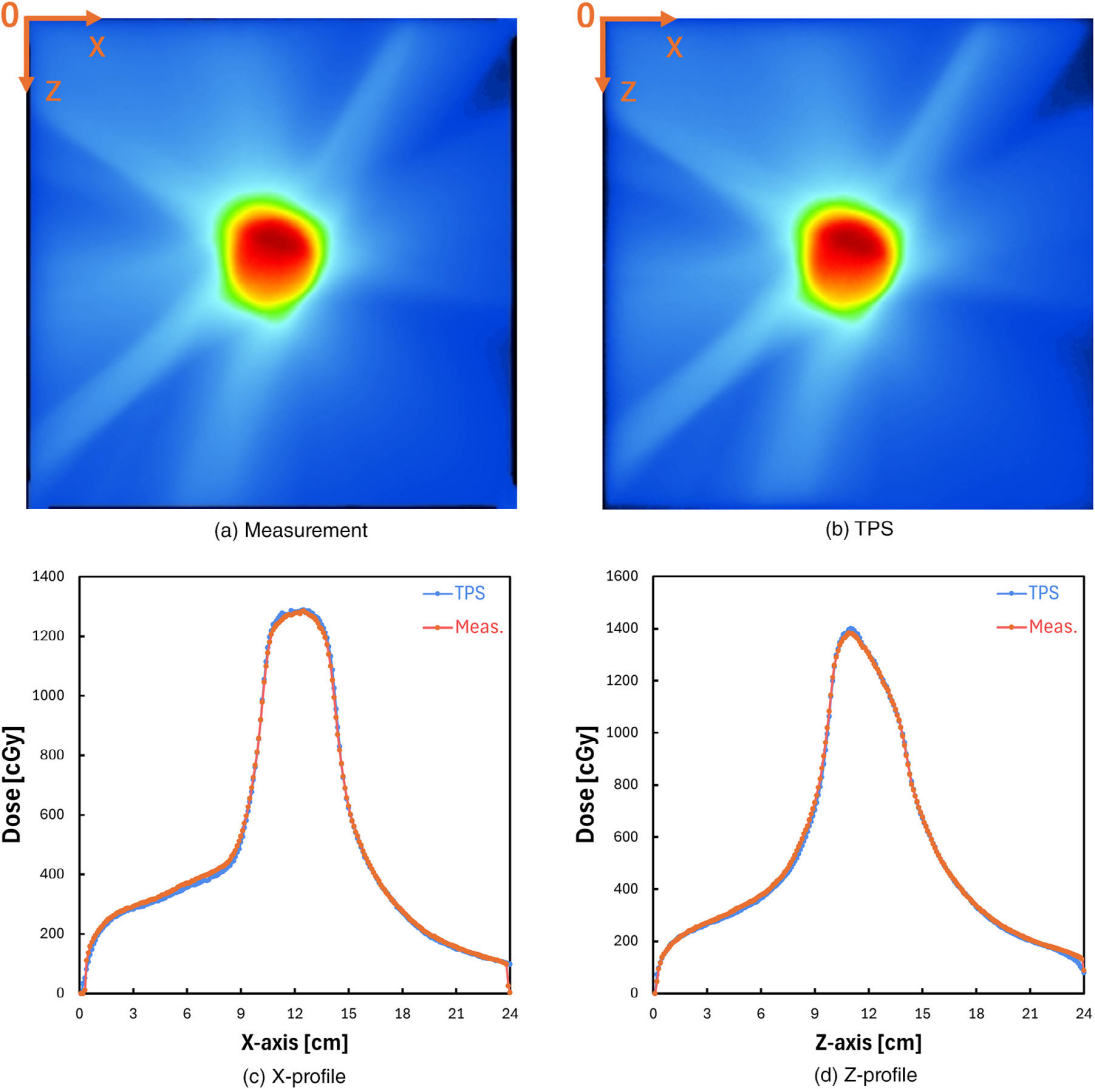
(a) A dose distribution measured for a clinical stereotactic lung VMAT case in the axial plane. (b) A dose distribution planned for a clinical stereotactic lung VMAT case in the axial plane. (c) *X*‐axis dose profile comparison between measurement and calculation. (d) *Z*‐axis dose profile comparison between measurement and calculation.

Figure 10a,b shows measured and planned dose distributions on an axial plane for a 6 MV FFF beam for the stereotactic lung VMAT. Figure 10c,d compares measured and planned dose distributions in the *X* and *Z* directions, respectively. The 2D gamma passing rates by using gamma analysis criteria of 3%/2 mm, 2%/2 mm, and 1%/1 mm with a dose threshold of 10% were observed to be 99.91%, 99.70%, and 91.04%, respectively. No corrections were made to these dose distributions in terms of field sizes, dose rates, and angles of incidence.

**FIGURE 11:**
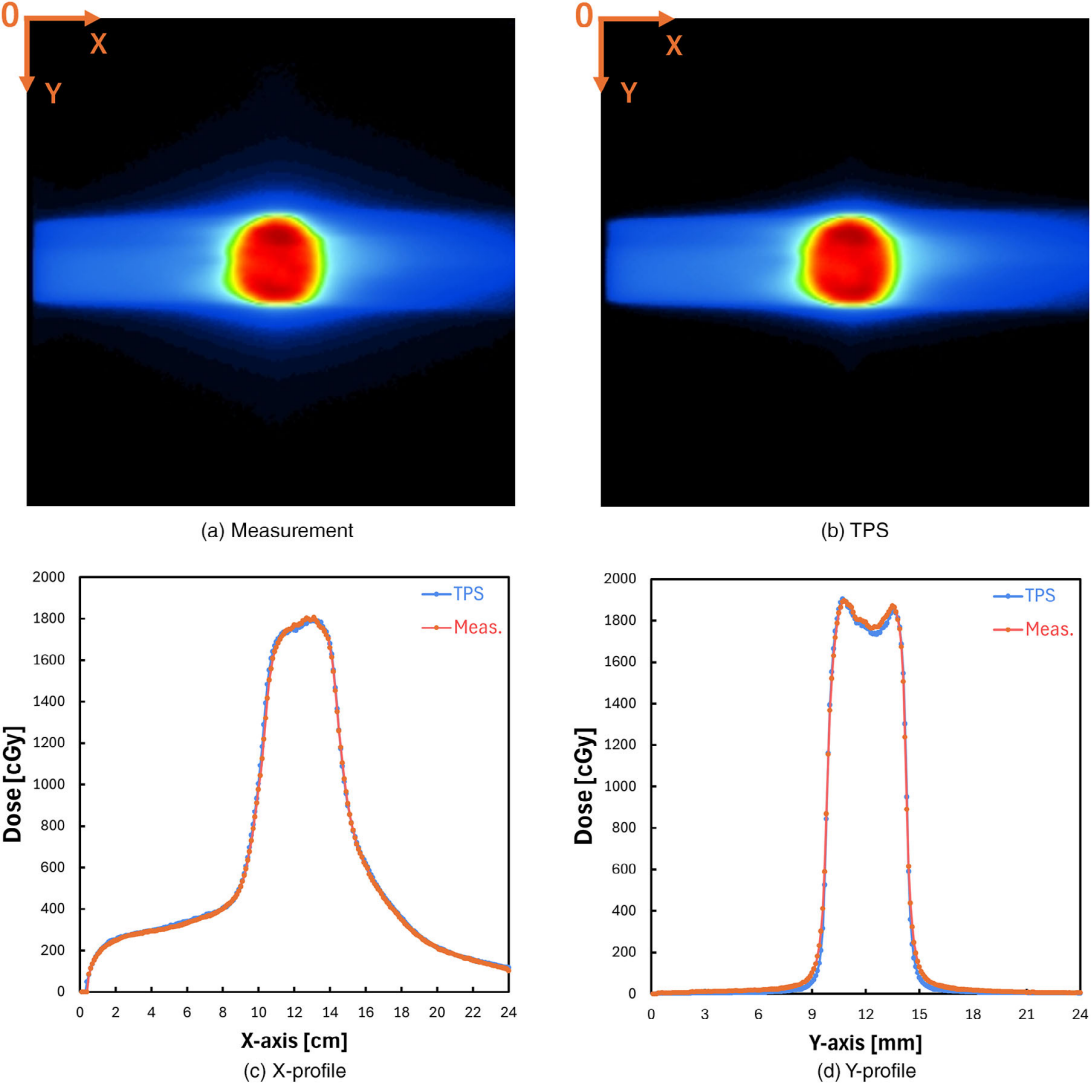
(a) A dose distribution measured for a clinical stereotactic lung VMAT case in the coronal plane. (b) A dose distribution planned for a clinical stereotactic lung VMAT case in the coronal plane. (c) *X*‐axis dose profile comparison between measurement and calculation. (d) *Y*‐axis dose profile comparison between measurement and calculation.

Figure 11a,b shows measured and planned dose distributions on a coronal plane for a 6 MV beam for the stereotactic lung VMAT, respectively. Figure 11c,d compares measured and planned dose distributions in the *X* and *Y* directions, respectively. Once again, the measured and calculated results showed good agreement. The 2D gamma passing rates by using gamma analysis criteria of 3%/2 mm, 2%/2 mm, and 1%/1 mm with 10% for the threshold were observed to be 100.00%, 99.90%, and 95.57%, respectively. No corrections were made to these dose distributions in terms of field size, dose rate, and angle of incidence.

Evaluation results of the plastic scintillation plate dosimeter and the reports of commercial array detectors are summarized in Table 2. Total measurement uncertainty is 2.4% (Linearity: 0.4%, Reproducibility: 0.062%, Dose rate dependence: 2.0%, Temperature dependence: 0.36%/6°C, Angular dependence: 1.3%, Energy dependence: −2.1%, +1.4%).

**TABLE 2:** Comparison of dose characteristics between the developed dosimeter and other commercial dosimeters.

	Linearity (Figure 2)	Reproducibility (Figure 3)	Dose rate dependence (Figure 4)	Temperature dependence (Figure 5)	Angular dependence (Figure 6)	Energy dependence (Table 1)
Plastic scintillation plate dosimeter	0.6% (1–1000 MU) 0.4% (10–1000 MU)	±0.062%	5.0% (45–1300 MU/min) 2.0% (160–1900 MU/min)	Camera: 0.06%/°C Scintillator: 0.2%/°C [18]	±1.3%	−2.1%, +1.4%
MapCHECK	0.5% [8] (10–300 MU)	±0.05% [9]	−1% [11] (400–2400 MU/min)	0.54%/°C [9]	−0%, +40% [10]	–
ArcCHECK	0.4% [12] (10–500 MU)	±0.05% [12]	<1% [13] (20–1400 MU/min)	–	−5%, +9% [12]	–
Delta4	0.25% [14] (43–863 MU)	±0.1% [14]	−0.1% [15] (109–1700 MU/mi)	0.4%/°C [14]	−0%, +8% [14]	–
MatriXX	0.6% [16] (1–3000 MU)	±0.1% [17]	−0.3% [16] (∼2400 MU/min)	0.3%/°C (Calculation from k_TP_) [20]	+19% [16]	–

## 4 DISCUSSION

A maximum linearity error of 0.6% was observed at a dose of 2 MU, and the error gradually decreased as the dose increased. The pulse frequency of the Elekta 6 MV beams giving the maximum dose rate is typically 400 Hz. When the maximum dose rate was 400 MU/min, a simple calculation indicates that a single pulse delivers a dose of 1/60 MU. The uncertainty in the delivered dose can be due to the amount of dose per pulse, which in our case is 1/60 MU. In other words, a relative error of 0.83% can be expected when a dose of 2 MU is delivered. This relative error would decrease proportionally as the delivered dose increases. This may explain the error profiles observed in the linearity test. For 10–1000 MU, the linearity error is 0.4%. The linearity error of our scintillation plate dosimeter is comparable to those of several array detectors: 0.5% (10–300 MU) for MapCHECK,^8^ 0.4% (10–500 MU) for ArcCHECK,^12^ 0.25% (43–863 MU) for Delta4,^14^ and 0.6% (1–3000 MU) for MatriXX.^16^

The dose reproducibility test showed that the coefficient of variation was 0.062%, which is also comparable to those of the above array detectors: 0.05% for MapCHECK,^9^ 0.05% for ArcCHECK,^12^ 0.1% for Delta4,^14^ and 0.1% for MatriXX.^17^

However, the dose rate dependency test for our scintillation plate system showed a drop of 5.0% at 45 MU/min, compared with that at 1300 MU/min for the 6 MV FFF beams, and 2.0% at 160 MU/min, compared with that at 1900 MU/min with 10 MV FFF beams. These values are larger than those for the other array detectors: 1% (400–2400 MU/min) for MapCHECK,^11^ 1% (20–1400 MU/min) for ArcCHECK,^13^ 0.1% (109–1700 MU/min) for Delta4,^15^ and 0.3% (up to 2400 MU/min).^16^ The larger variation in the scintillation plate dosimeter may be due to the camera's insensitivity. At the lowest dose rate, the number of photons captured by the image sensor is limited to only a few. Under these low‐light conditions, the linear relationship between light intensity (dose) and the camera's output signal may have been compromised. The insensitivity of optical cameras has been documented in the literature on measurements of 2D scintillator arrays.^19^

Our dosimeter comprises a scintillator and camera, both of which depend on the temperature. The temperature dependence of the plastic scintillator was reported to be 0.2%/°C at room temperature.^18^ On the other hand, the temperature dependence of our CMOS camera was evaluated to be approximately 0.06%/°C in our study. Note that this temperature is the value of the temperature sensor attached to the image sensor, but its accuracy has not been verified. The CMOS sensor temperature rises from 24°C to 27°C within approximately five minutes after measurement begins. Assuming a temperature change of 3°C during a QA session, the camera output is expected to increase by 0.18%. Since the camera temperature can be monitored, real‐time corrections to the camera output signal can be applied as needed. The temperature dependence of other array detectors has been reported as 0.54%/°C for MapCHECK^9^ and 0.4%/°C for Delta4.^14^ No publications were found for MatriXX, but the use of the k_TP_ correction factor for an ionization chamber led to 0.3%/°C^20^ under standard atmospheric conditions.

The angular dependence of our system showed a variation of ± 1.3%. This variation is far smaller than those of the other array detectors: 0 to 40% for MapCHECK,^10^ −5% to 9% for ArcCHECK,^12^ 0 to 8% for Delta4,^14^ and 0 to 19% for MatriXX.^16^ Although there is a significant dependence on the angle, each of the other array detectors reduces this impact by installing an angle sensor on a gantry as a compensator. The angular dependence arises because the TPS does not accurately account for the effects of cavities inside the detector or the presence of high‐atomic‐number materials. In contrast, the scintillation plate system has a density of 1.04 g/cm^3^, close to that of water, and a similar elemental composition, resulting in minimal intrinsic angular dependence. Consequently, the proposed system does not require a gantry angle sensor.

The x‐ray energy dependence of our system showed a variation from −2.1% to +1.4% among five different energies, in reference to 6 MV x‐ray beams. This correction factor can be used for dose measurements at each energy level. Notably, the energy dependency results for array detectors are not available in the literature. Therefore, it is assumed that energy calibration must be performed prior to dose measurements for each energy with these array detectors.

The limitations of this study include the following. First, a single linac was employed for the test. Therefore, the data may contain the characteristics of the linac used. Second, the Monaco TPS was used for dose calculation with an uncertainty of 0.5%. Third, the PMMA and scintillator were not optically bonded, and there was a small air gap of less than 0.1 cm between them, which may have affected the dose normalization results from the TPS calculation with a dose grid size of 2.0 mm. The calculation accuracy may be improved by adjusting the density of PMMA in the TPS.

In this study, we have put to practical use a scintillator‐based dose distribution measurement device for VMAT and conducted a comprehensive assessment of its characteristics. To enable absolute dose measurements, further research is required, including software corrections and the development of plastic scintillators that accurately respond to x‐ray intensity. The current dosimeter can be used for measuring relative distributions in the patient‐specific QA.

In the evaluation of VMAT for lung stereotactic radiotherapy, it was shown that this dosimeter has the potential to be used for patient QA with no corrections. The scintillator‐based dosimeter developed has the potential to be a useful tool in the field of radiation therapy, with its high resolution, wide measurement range, and repeated dose measurements without touching the device.

## 5 CONCLUSION

The combination of a scintillation plate dosimeter and a CMOS camera indicated that the dosimetry characteristics were favorable. It may be more widely used for dose QA in radiotherapy, particularly for smaller‐field stereotactic VMAT, because of its water equivalence and higher spatial resolution. The authors believe that these characteristics are major differentiators from array detectors that use ionization chambers or diodes.

## CONFLICT OF INTEREST STATEMENT

The authors declare no conflicts of interest.
